# Supplementary material for: The circadian clock and darkness control natural competence in cyanobacteria
Source: Nat Commun. 2020 Apr 3;11:1688. doi: 10.1038/s41467-020-15384-9 (PMC7125226; doi:10.1038/s41467-020-15384-9)
Supplement: Supplementary file 1 — Supplementary Information [file 41467_2020_15384_MOESM1_ESM.pdf]

Supplementary Information

The circadian clock and darkness control natural competence in cyanobacteria

Taton et al.

Supplementary Figures

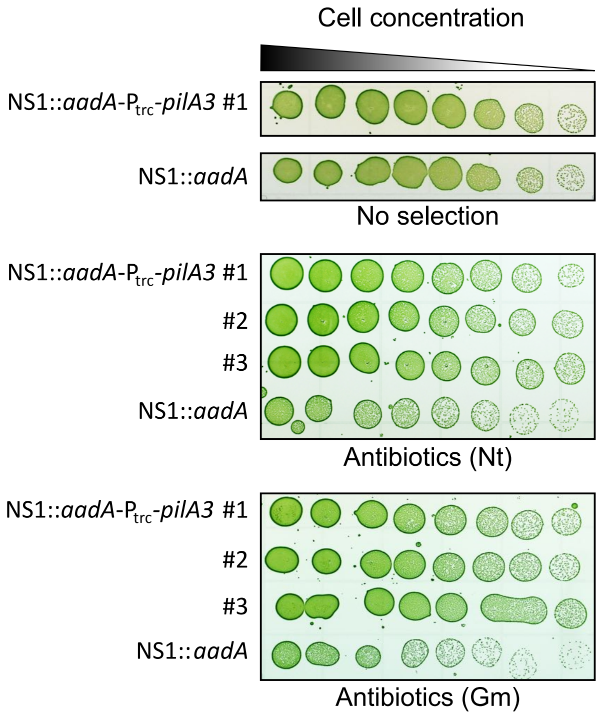

Supplementary Fig. 1 Overexpression of *pilA3* results in increased transformation. Semi-quantitative transformation assays performed on a *pilA3* overexpression strain. The assays were performed on 3 independent clones with plasmids that carry different antibiotic resistance genes for gentamycin (Gm) or nourseothricin (Nt) and target distinct neutral sites (NS) in the *S. elongatus* chromosome. The control strain marked NS1::*aadA* is engineered to carry only antibiotic resistance, and no *pilA3* gene, at a neutral site.

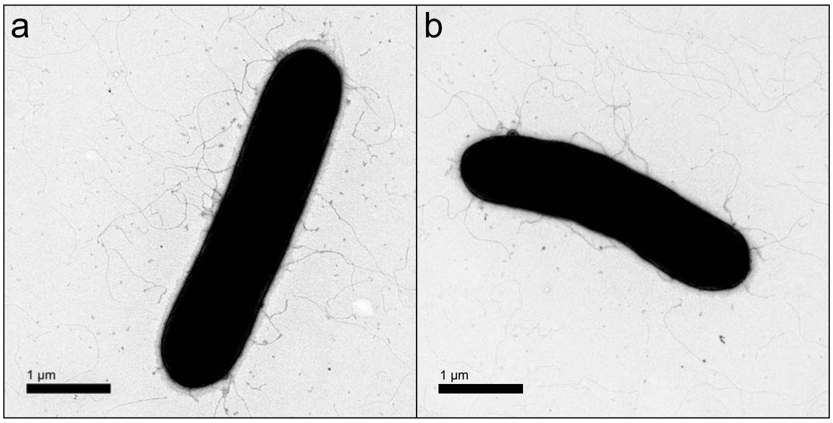

Supplementary Fig. 2 *S. elongatus* remains piliated upon deletion of *pilA3* and *pilW* (AMC2547) or *rntA* and *rntB* (AMC2543). a Transmission electron microscopy (TEM) pictures of strain AMC2547. **b** TEM pictures of strain AMC2543.

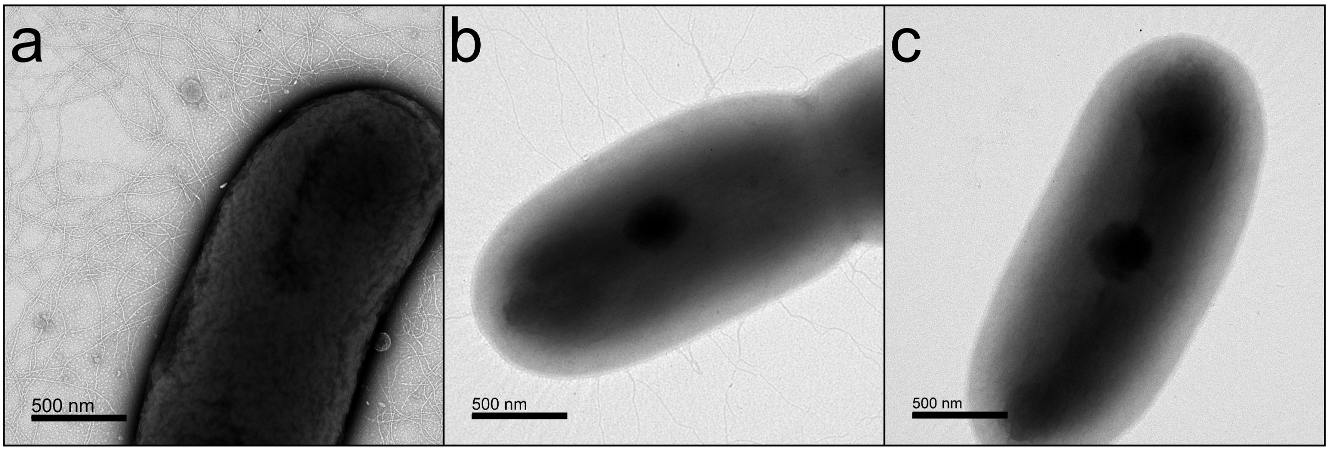

Supplementary Fig. 3 Transmission electron micrographs taken at ZT 6 of cells grown in LD and subjected to repeated washes to denude the cells of their pili. a Cells were collected from the growing flask at ZT 6 and fixed for electron microscopy. **b** Cells were collected from the growing flask at ZT 0, washed twice, resuspended in fresh medium and incubated with the growing flask until ZT 6, when they were fixed for electron microscopy. **c** Cells were subjected to the same procedure as in (**b**) but the washing steps were repeated at ZT 6 prior to fixation for electron microscopy.

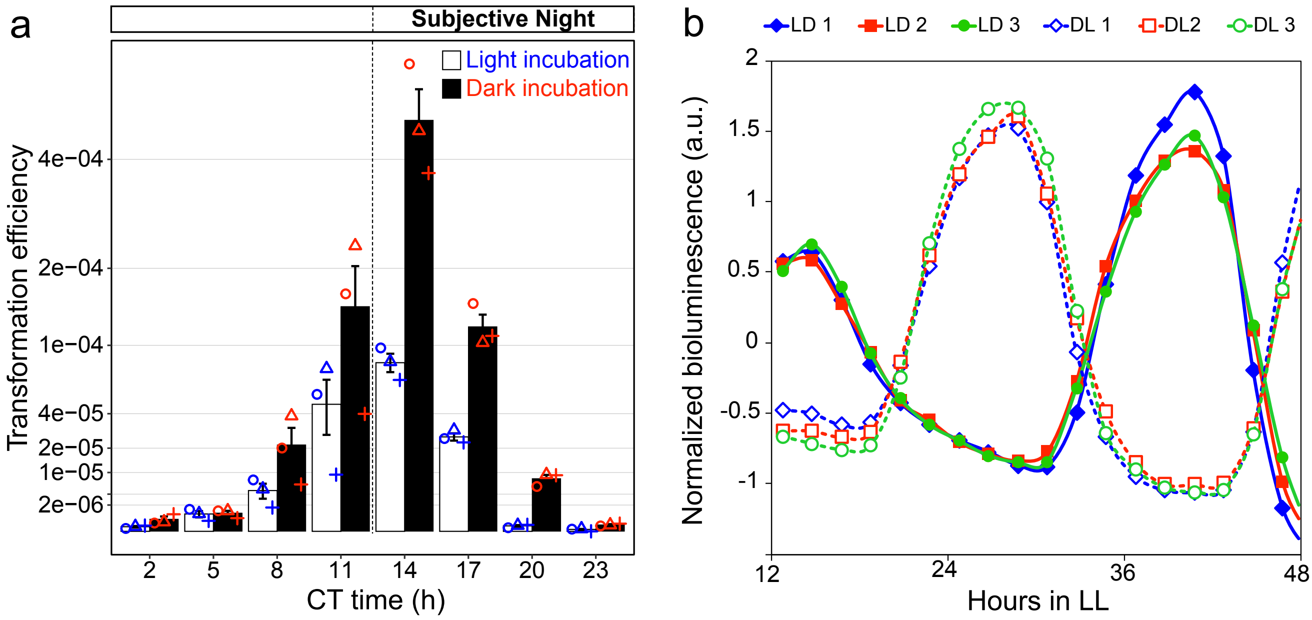

Supplementary Fig. 4 Competence is circadian and induced by darkness. a Transformation efficiency in *S. elongatus* circadian reporter strain AMC1300 over a 24-h circadian cycle. Efficiencies were calculated as the number of antibiotic resistant colonies per CFU without selection upon transformation of 3 biologically independent cultures (circles, triangles, and plus signs) and plotted as mean values ± standard error of means (SEM) on a square-root scale. **b** Circadian rhythmicity of gene expression in cultures of *S. elongatus* AMC1300 used for the transformation assays. Bioluminescence from strain AMC1300 is produced by a P_{kaiB}-*luxAB*/P_{psbA}-*luxCDE* reporter system. LD or DL show the order of 12-h light and dark entrainment prior to the continuous-light monitoring period. Source data are provided as a Source Data file.

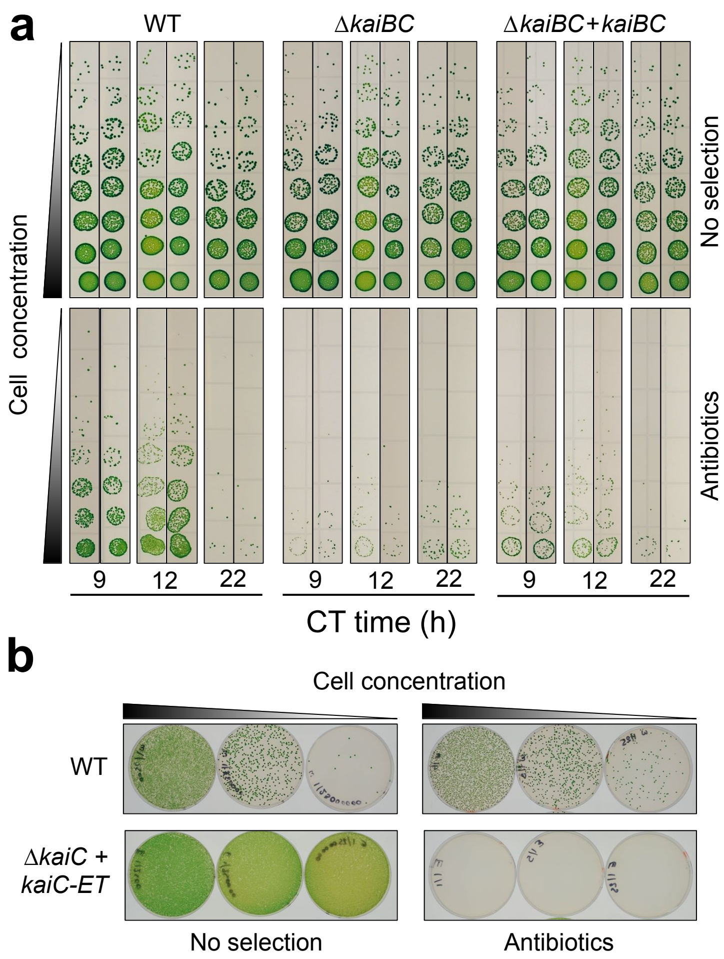

Supplementary Fig. 5 Circadian rhythmicity of transformation is controlled by the circadian oscillator. a Transformation assays performed at key CT points on a WT strain, a *kaiBC*-null strain and the *kaiBC*-null strain complemented with the *kaiBC* locus at a neutral site. WT *S. elongatus* PCC 7942 served as a positive control. The assays were performed in triplicate (duplicates are shown) and yielded identical results. Incomplete complementation is probably due to differences in expression levels from the neutral site relative to the native locus. **b** Transformation experiment, during which cells were incubated with eDNA in low light for 16 h, that illustrates the loss of transformation in a mutant strain that express the phosphomimetic allele *kaiC-ET*. No transformants were detected for the *kaiC-ET* mutant even at high concentrations of input cells. Source data are provided as a Source Data file.

Supplementary Tables

Supplementary Table 1 Plasmids and strains used in this study

Strain or Plasmid	Description / Genotype	Antibiotic resistance	Reference
**Devices, expression vectors**			
pCVD026	pBR322 origin of replication and base of mobilization carried on a CYANO-VECTOR donor plasmid	Ap	[4]
pCVD003	Km^R gene cassette carried on a CYANO-VECTOR donor plasmid	Ap, Km	[4]
pCVD007	Cm^R resistance gene cassette carried on a CYANO-VECTOR donor plasmid	Ap, Cm	[4]
pAM2991	Expression vector targeting *S. elongatus* NS1 with a Sp and Sm resistance gene cassette and an IPTG inducible *lacI*^{q}-P$_{trc}$ promoter system, NS1::(*aadA-lacI*^{q}-P$_{trc}$).	Sp+Sm	[5]
pAM5433	Expression vector for *S. elongatus* NS3 harboring a Gm resistance gene cassette and an IPTG inducible *lacI*^{q}-P$_{trc}$ promoter system, NS3::(*aacC1-lacI*^{q}-P$_{trc}$).	Gm	Laboratory collection*
pAM5467	NS2::(*aacC1*-P$_{T7}$-*yfp*)	Gm	[6]
pAM5470	NS1::(*aadA*-P$_{conII*}$- RSWB-*T7RNAP*)	Sp+Sm	[6]
**Insertional knockout plasmids**			
8S9-J3	*S. elongatus* gDNA library clone with a Tn5 insertion in *pilB*	Km	[7]
8S7-F2	*S. elongatus* gDNA library clone with a Tn5 insertion in *pilD*	Km	[7]
2E8-E4	*S. elongatus* gDNA library clone with a Mu insertion in *pilA2*	Cm	[7]
1B2-E-C1	*S. elongatus* gDNA library clone with a Tn5 insertion in *comEA*	Km	[7]
8S35-A8	*S. elongatus* gDNA library clone with a Tn5 insertion in *comEC*	Km	[7]
2E8-EEE2	*S. elongatus* gDNA library clone with a Mu insertion in *rntA*	Cm	[7]
2E8-JJJ10	*S. elongatus* gDNA library clone with a Mu insertion in *rntB*	Cm	[7]
8S36-N7	*S. elongatus* gDNA library clone with a Tn5 insertion in *pilA3*	Km	[7]
**Plasmids for transformation assays**			
pAM5328	NS3::*aacC1*	Gm	[8]
pAM5329	NS1::*aadA*	Sp+Sm	[8]
pAM5544	NS2::*nat7942*	Nt	(This study)
pAM5554	NS3::*nat7942*	Nt	(This study)
**Deletion knockout plasmids**			
pAM5545	Δ(*pilA3-pilW*)::*cat7942*	Cm	(This study)
pAM5546	Δ(*rntA-rntB*)::*aphI*	Km	(This study)
**Complementation plasmids**			
pAM4645	NS2::(*nat7942*-P*kaiBC-kaiBC*)	Nt	[9]
pAM5547	NS1::(*aadA-lacI*^{q}-P$_{trc}$-*pilA3*)	Sp+Sm	(This study)
pAM5548	NS3::(*aacC1-lacI*^{q}-P$_{trc}$-*pilW*)	Gm	(This study)
pAM5549	NS3::(*aacC1-lacI*^{q}-P$_{trc}$-*rntA*)	Gm	(This study)

pAM5550	NS2::(*aacC1*-P_{T7}-*rntB*)	Gm, Tc	(This study)
pAM5551	NS2::(*aacC1*-P_{T7}-*rntA-rntB*)	Gm, Tc	(This study)
pAM5552	NS1::(*aadA-lacI*^{q}-P_{trc}-*sigF2*)	Sp+Sm	(This study)
**Strains**			
AMC06	Wild-type strain of *Synechococcus elongatus* PCC 7942		Laboratory collection
LIB1.0	*S. elongatus* PCC 7942 RbTn-Seq library	Km	[10]
AMC_705	Δ*kaiBC*, NS2::(*cat*-P*kaiBC-luc*)	Cm	[11]
AMC1300	NS1::(*aadA*-P_{kaiB}-*luxAB*), NS2::(*aph1*-P_{psbAI}-*luxCDE*)	Km, Sp+Sm	[12]
AMC2106	Δ*kaiC*, NS2::(*aph1*-P_{purF}-*luc*), NS1::(*aadA-lacI*^{q}-P_{trc}-*kaiC*)	Km, Sp+Sm	[13]
AMC2109	Δ*kaiC*, NS2::(*aph1*-P_{purF}-*luc*), NS1::(*aadA-lacI*^{q}-P_{trc}-*kaiC-ET*)	Km, Sp+Sm	[13]
AMC2110	Δ*kaiC*, NS2::(*aph*1-P_{purF}-*luc*), NS1::(*aadA-lacI*^{q}-P_{trc}-*kaiC-SE*)	Km, Sp+Sm	[13]
AMC2543	Δ(*rntA-rntB*)::*aphI*	Km	(This study)
AMC2544	Δ(*rntA-rntB*)::*aphI*, NS3::(*aacC1-lacI*^{q}-P_{trc}-*rntA*)	Km, Gm	(This study)
AMC2545	Δ(*rntA-rntB*)::*aphI*, NS2::(*aacC1*-P_{T7}-*rntB*), NS1::(*aadA*-P_{conII*}- RSWB-T7RNAP)	Km, Gm, Sp+Sm	(This study)
AMC2546	Δ(*rntA-rntB*)::*aphI*, NS2::(*aacC1*-P_{T7}-*rntA-rntB*), NS1::(*aadA*-P_{conII*}- RSWB-T7RNAP)	Km, Gm, Sp+Sm	(This study)
AMC2547	Δ(*pilA3-pilW*)::*cat7942*	Cm	(This study)
AMC2548	Δ(*pilA3-pilW*)::*cat7942*, NS1::(*aadA-lacI*^{q}-P_{trc}-*pilA3*)	Cm, Sp+Sm	(This study)
AMC2549	Δ(*pilA3-pilW*)::*cat7942*, NS3::(*aacC1*-l*acI*^{q}-P_{trc}-*pilW*)	Cm, Gm	(This study)
AMC2550	Δ(*pilA3-pilW*)::*cat7942*, NS1::(*aadA-lacI*^{q}-P_{trc}-*pilA3*), NS3::(*aacC1-lacI*^{q}-P_{trc}-*pilW*)	Cm, Gm Sp+Sm	(This study)
AMC2551	*sigF2*::Mu(5A4-F6)	Cm	(This study)
AMC2552	*sigF2*::Mu(5A4-F6), NS1::(*aadA-lacI*^{q}-P_{trc}-*sigF2*)	Cm, Sp+Sm	(This study)

Abbreviations for antibiotics: Cm, chloramphenicol; Gm, gentamycin; Km, kanamycin; Nt, nourseothricin; Sm, streptomycin; Sp, spectinomycin. * Plasmid constructed by Susan E. Cohen

Supplementary Table 2 | List of primers used for RT-qPCR analyses

Name	Target gene	Sequence (5'-3')
rnpB_qPCR_F1	*rnpB*	TGAGGAGAGTGCCACAGAAACA
rnpB_qPCR_R1	*rnpB*	ACCCTTACCTGTGCCTGCAA
dprA_qPCR_F2	*dprA*	AAGCCGGTTACACCGTGATT
dprA_qPCR_R2	*dprA*	TGATGGGCTTCCGTATCCA
comEA_qPCR_F	*comEA*	CCCTGGCTTTGCCTATCGT
comEA_qPCR_R	*comEA*	AGCGCTAGGCCGACCATAT
pilA3_qPCR_F	*pilA3*	ATGCCCAAGCCCAGTTAATG
pilA3_qPCR_R	*pilA3*	ACAGGTGCGATTGGTTCGTT
rntA_qPCR_F	*rntA*	TGGCCAGAACCCTCAAAGTG
rntA_qPCR_R	*rntA*	TCGGGTGTTGTTGCACGAT
pilQ_qPCR_F	*pilQ*	CGGGAGCGAGTTGCTAAGAT
pilQ_qPCR_R	*pilQ*	CGAAAGAGGGAACCGATCAG

Supplementary References

1 Markson, J. S., Piechura, J. R., Puszynska, A. M. & O'Shea, E. K. Circadian control of global gene expression by the cyanobacterial master regulator RpaA. *Cell* **155**, 1396-1408 (2013).

2 Vijayan, V., Zuzow, R. & O'Shea, E. K. Oscillations in supercoiling drive circadian gene expression in cyanobacteria. *Proc Natl Acad Sci U S A* **106**, 22564-22568 (2009).

3 Piechura, J. R., Amarnath, K. & O'Shea, E. K. Natural changes in light interact with circadian regulation at promoters to control gene expression in cyanobacteria. *Elife* **6**, R106 (2017).

4 Taton, A. *et al.* Broad-host-range vector system for synthetic biology and biotechnology in cyanobacteria. *Nucleic Acids Res* **42**, e136 (2014).

5 Ivleva, N. B., Bramlett, M. R., Lindahl, P. A. & Golden, S. S. LdpA: a component of the circadian clock senses redox state of the cell. *EMBO J* **24**, 1202-1210 (2005).

6 Roulet, J. *et al.* Development of a cyanobacterial heterologous polyketide production platform. *Metab Eng* **49**, 94-104 (2018).

7 Chen, Y., Holtman, C. K., Taton, A. & Golden, S. S. Functional analysis of the *Synechococcus elongatus* PCC 7942 genome 119-137 (Springer Netherlands, 2012).

8 Taton, A., Ma, A. T., Ota, M., Golden, S. S. & Golden, J. W. NOT gate genetic circuits to control gene expression in cyanobacteria. *ACS Synth Biol* **6**, 2175-2182 (2017).

9 Cohen, S. E., Erb, M. L., Selimkhanov, J., Dong, G., Hasty, J., Pogliano, J., Golden, S. S. Dynamic localization of the cyanobacterial circadian clock proteins. *Curr Biol* **24**,1836-1844 (2014).

10 Rubin, B. E. *et al.* The essential gene set of a photosynthetic organism. *Proc Natl Acad Sci U S A* **112**, E6634-6643 (2015).

11 Ditty, J. L., Canales, S. R., Anderson, B. E., Williams, S. B. & Golden, S. S. Stability of the Synechococcus elongatus PCC 7942 circadian clock under directed anti-phase expression of the kai genes. *Microbiology* **151**, 2605-2613 (2005).

12 Chen, Y. *et al.* A novel allele of *kaiA* shortens the circadian period and strengthens interaction of oscillator components in the cyanobacterium *Synechococcus elongatus* PCC 7942. *Journal of bacteriology* **191**, 4392-4400 (2009).

13 Paddock, M. L., Boyd, J. S., Adin, D. M. & Golden, S. S. Active output state of the *Synechococcus* Kai circadian oscillator. *Proc Natl Acad Sci U S A* **110**, E3849-3857 (2013).
