# Supplementary material for: The circadian clock and darkness control natural competence in cyanobacteria
Source: Nat Commun. 2020 Apr 3;11:1688. doi: 10.1038/s41467-020-15384-9 (PMC7125226; doi:10.1038/s41467-020-15384-9)
Supplement: Supplementary file 3 — Description of Additional Supplementary Files [file 41467_2020_15384_MOESM3_ESM.pdf]

Description of Additional Supplementary Files

File Name: Supplementary Data 1
Description: The results of the RB-TnSeq analysis and the transcriptomics discussed in this study. For each gene that is not essential for the survival of S. elongatus under laboratory conditions, this table provides: locus tag, genomic location, gene/locus name, gene description, the results of the RB-TnSeq analysis including fitness estimate and associated statistics under selective vs control conditions (Fitness, Std error, T value, P value, P value model, P Holm model, P fdr model, High confidence), and published transcriptomics data of S. elongatus during a circadian time course and upon shading.

File Name: Supplementary Data 2
Description: The raw data (read counts for each barcode or mutant strain, “all.poolcount.txt”), the R scripts (“Transformation_Screen_AT051018.Rmd” and Transformation_Screen_AT051018.nb.html) to analyze these data, the annotation of each locus in the S. elongatus genome (locus tag, gene name, gene location, and gene description, “genes.tab”), and the result of the analysis (Fitness, Std error, T value, P value, P value model, P Holm model, P fdr model, “Transformation_Screen_Estimates_AT051018.csv”). In addition, this archive also includes a description of the samples (“Transformation_screen_AT051018.csv”) as required by the R-scripts and a document (“READ_ME.txt”) that lists and describes each file within the archive.
