# Supplementary material for: The circadian clock and darkness control natural competence in cyanobacteria
Source: Nat Commun. 2020 Apr 3;11:1688. doi: 10.1038/s41467-020-15384-9 (PMC7125226; doi:10.1038/s41467-020-15384-9)
Supplement: Supplementary file 6 — Reporting Summary [file 41467_2020_15384_MOESM6_ESM.pdf]

nature research

Corresponding author(s): Susan S. Golden

Last updated by author(s): Feb 28, 2020

Reporting Summary

Nature Research wishes to improve the reproducibility of the work that we publish. This form provides structure for consistency and transparency in reporting. For further information on Nature Research policies, see Authors & Referees and the Editorial Policy Checklist .

Statistics

For all statistical analyses, confirm that the following items are present in the figure legend, table legend, main text, or Methods section.

n/a	Confirmed	
☐	☒	The exact sample size (*n*) for each experimental group/condition, given as a discrete number and unit of measurement
☐	☒	A statement on whether measurements were taken from distinct samples or whether the same sample was measured repeatedly
☐	☒	The statistical test(s) used AND whether they are one- or two-sided<br>*Only common tests should be described solely by name; describe more complex techniques in the Methods section.*
☒	☐	A description of all covariates tested
☐	☒	A description of any assumptions or corrections, such as tests of normality and adjustment for multiple comparisons
☐	☒	A full description of the statistical parameters including central tendency (e.g. means) or other basic estimates (e.g. regression coefficient) AND variation (e.g. standard deviation) or associated estimates of uncertainty (e.g. confidence intervals)
☐	☒	For null hypothesis testing, the test statistic (e.g. *F*, *t*, *r*) with confidence intervals, effect sizes, degrees of freedom and *P* value noted<br>*Give P values as exact values whenever suitable.*
☒	☐	For Bayesian analysis, information on the choice of priors and Markov chain Monte Carlo settings
☒	☐	For hierarchical and complex designs, identification of the appropriate level for tests and full reporting of outcomes
☒	☐	Estimates of effect sizes (e.g. Cohen's *d*, Pearson's *r*), indicating how they were calculated

Our web collection on statistics for biologists contains articles on many of the points above.

Software and code

Policy information about availability of computer code

Data collection	The barcodes were quantified in perl 5v18 using custom codes published previously. A reference is provided in the methods section.<br>Colony counts were collected using ImageJ 1.52k.
Data analysis	The RB-TnSeq, transcriptomics, transformation efficiency and RT-qPCR data were analyzed in R version 3.6.0. Custom R scripts for the RB-TnSeq analysis were published previously and references provided in the methods section.<br>BLASTp searches were conducted using BioBIKE (http://biobike.csbc.vcu.edu/).<br>Phylogenetic analyses were performed in MEGA 7.0, from an alignment produced with MUSCLE as implemented in MEGA 7.0 and Gblocks 0.91b.

For manuscripts utilizing custom algorithms or software that are central to the research but not yet described in published literature, software must be made available to editors/reviewers. We strongly encourage code deposition in a community repository (e.g. GitHub). See the Nature Research guidelines for submitting code & software for further information.

Data

Policy information about availability of data

All manuscripts must include a data availability statement. This statement should provide the following information, where applicable:
- Accession codes, unique identifiers, or web links for publicly available datasets
- A list of figures that have associated raw data
- A description of any restrictions on data availability

The RB-TnSeq results and previously published transcriptomics analyses underlying Figs. 1a, 2b, 3a, and 3b are provided in the Supplementary Ddata 1 file. The raw data to perform the RB-TnSeq analysis are provided in the Supplementary Ddata 2 file. Descriptions of plasmids, strains and RT-qPCR primers are available as supplementary tables; additional supporting materials are provided as supplementary figures.. The data underlying Figs. 1a, c, 2a-c, 3a-d, 4a-c, 5a-d3d, 4c, 5, and Supplementary Figs. 4a, b and 5a are provided in the Source data Data file. Descriptions of plasmids, strains and RT-qPCR primers are available as supplementary tables; additional supporting materials are provided as supplementary figures. All other data produced and/or analyzed to support the findings of the study are

available from the corresponding author upon request.

Field-specific reporting

Please select the one below that is the best fit for your research. If you are not sure, read the appropriate sections before making your selection.

- [x] Life sciences
- [ ] Behavioural & social sciences
- [ ] Ecological, evolutionary & environmental sciences

For a reference copy of the document with all sections, see nature.com/documents/nr-reporting-summary-flat.pdf

Life sciences study design

All studies must disclose on these points even when the disclosure is negative.

Sample size

- No statistical method was used to predetermine the number of colonies collected for the RB-TnSeq analysis. For each condition and experiment we aimed to collect about 200,000 colonies, corresponding roughly to 100 times the number of non-essential genes in Synechococcus elongatus. We estimated that 70,000 to 250,000 colonies per condition were collected. The library at T0s accounted for 149,277 strains across replicates of which 127,180 were located within coding sequences, thereby corresponding to an average of 66 strains per non-essential coding sequence. The analysis revealed that across replicates 82% and 93% of the strains in the library were sampled in treatment and control conditions, respectively. Taking into account the high library insertion density and the concurrent results obtained for replicates we concluded that the sample sizes were sufficient to determine the genetic basis of natural transformation in S. elongatus.
- To determine transformation efficiencies, colony counts were performed at dilutions that resulted in the maximum number of isolated colonies per well. Colony counts were obtained from 2 wells for the selective (with antibiotics) condition and one well under non-selective control condition (which remains relatively constant among samples). We calculated that an average of 73 colonies per well were counted for the experiment presented in figure 5, for example. From the incremental changes in transformation efficiency of S. elongatus during a circadian time course and small differences between replicates, we conclude that the sample sizes were sufficient to obtain accurate measures of transformation efficiencies.

Data exclusions

As part of the RB-TnSeq analysis pipeline barcode reads and barcodes were curated as described in the methods section. To maximize the likelihood that a transposon insertion would result in the loss of function of the targeted gene and to follow pre-established guidelines:
- The barcodes located outside of the middle 80% of the annotated coding sequence were excluded from the analyses.
- Genes represented by less than three barcodes in different positions or less than 15 T0 reads across replicates were excluded from the analyses.

Replication

- To screen the Rb-TnSeq Library for genes involved in natural competence, 3 experiments were performed. The significance of a fitness score given to any particular gene was provided and takes into account the 3 experiments.
- All transformation assays were done in duplicate or triplicates from 2 or 3 independent clones.
- Quantitative transformation assays performed along a circadian time course were done in triplicate using independent cultures. The experiment whose results are presented in Figure 3d was repeated and the results presented as supplementary Figure 4. For the experiment whose results are presented in Figure 5, a prior experiment was performed and, although the experiment carried a few design flaws, it yielded similar results.
- RT-qPCRs were done using 3 biological replicates and 2 technical replicates.
- Electron photomicrographs were chosen as representatives of 5 to 10 pictures of cells selected randomly and prepared from 2 independent cultures for each time point. In addition, the experiment was repeated for ZT 0 – 6 and provided as supplementary information. (Preliminary EM observations also indicated the same finding.)

Randomization

Standard randomization procedures were not directly applicable or required for this study, but:
- We used a high-density library of randomly barcoded transposon mutants to determine the genes required for natural transformation.
- Experiments were carried out using independent cultures (in triplicate). For genetic backgounds other than the wild type, the triplicates derived from independent transformation events.
- Cells analyzed by electron microscopy were picked at random. Initially, the positions of 5 to 10 cells were recorded using a low magnification that would not allow the distinction of features, then each cell was photographed at higher magnification.

Blinding

Standard blinding procedures were not directly applicable or required for this study, but:
- The features or competence behaviors of the mutant strains could not be predicted before they were evaluated experimentally.
- Pictures taken to determine transformation efficiencies were manually curated to account for colony doublets (or small numbers of joined colonies), but the sample information and dilution factors were ignored during the process and colony counts were performed using the software ImageJ, thereby limiting subjective bias from the investigator.
- Electron micrographs were taken from cells selected at a low magnification that would not allow the distinction of the cell features.

Reporting for specific materials, systems and methods

We require information from authors about some types of materials, experimental systems and methods used in many studies. Here, indicate whether each material, system or method listed is relevant to your study. If you are not sure if a list item applies to your research, read the appropriate section before selecting a response.

Materials & experimental systems

n/a	Involved in the study
☒	☐ Antibodies
☒	☐ Eukaryotic cell lines
☒	☐ Palaeontology
☒	☐ Animals and other organisms
☒	☐ Human research participants
☒	☐ Clinical data

Methods

n/a	Involved in the study
☒	☐ ChIP-seq
☒	☐ Flow cytometry
☒	☐ MRI-based neuroimaging
